# Analysis of Game Performance Indicators during 2015–2019 World Padel Tour Seasons and Their Influence on Match Outcome

**DOI:** 10.3390/ijerph18094904

**Published:** 2021-05-04

**Authors:** Adrián Escudero-Tena, Bernardino Javier Sánchez-Alcaraz, Javier García-Rubio, Sergio J. Ibáñez

**Affiliations:** 1 Training Optimization and Sport Performance Research Group (GOERD), Sport Science Faculty, University of Extremadura, 10005 Caceres, Spain; adescuder@alumnos.unex.es (A.E.-T.); jagaru@unex.es (J.G.-R.); sibanez@unex.es (S.J.I.); 2 Department of Physical Activity and Sport, Faculty of Sport Sciences, University of Murcia, C/Argentina, s/n, 30700 Murcia, Spain

**Keywords:** racket sports, performance analysis, technical indicators, tactical indicators, paddle tennis

## Abstract

A better understanding of the demands of in-game competition demands may improve coaching strategies, training designs, and injury prevention programs. However, there is limited information regarding performance analysis in professional padel. This study aimed to analyse performance indicators and their influence on match outcomes regarding sex, tournament round, and set number. The sample contained 1070 sets from 532 matches of the 2016 to 2019 World Padel Tour seasons. Variables including sex, round, game result, stroke effectiveness, and break points were registered through systematic observation. A non-parametric approach was applied to evaluate differences between sex, match outcome, and tournament round. The results showed significant differences between winners and losers regarding sex in break points (male *d* = 2.13, *p* = 0.00; female *d* = 2.22, *p* = 0.00), smash winners (male *d* = 0.85, *p* = 0.00; female *d* = 0.69, *p* = 0.00), groundstroke winners (male *d* = 1.01, *p* = 0.00; female *d* = 1.18, *p* = 0.00), volley winners (male *d* = 1.08, *p* = 0.00; female *d* = 0.91, *p* = 0.00), and errors (male *d* = 0.76, *p* = 0.00; female *d* = 0.65, *p* = 0.00). Furthermore, differences in shot effectiveness between winners and errors increased in the last set of the match and in the last round of the tournament (*p* < 0.05). Therefore, shot effectiveness seems to be a key factor in professional padel that distinguishes between winning and losing players. Such knowledge may have implications in the design of appropriate game strategies and specific training sessions to improve performance and to prevent sport injuries.

## 1. Introduction

Padel is a recent sport [1], which is practised in pairs (two vs. two) on a 20 × 10 m court surrounded by walls or glass and metal fences, which allow the bouncing of the ball, and is scored like tennis [2]. In recent years, there has been an enormous growth in the number of players and courts, as it is now practised in more than 40 countries around the world [3,4]. Furthermore, a professional padel circuit has been developed (World Padel Tour), with tournaments in several countries. Padel practice has significant advantages compared with other racket sports, which renders it a powerful tool for health promotion, namely: high technical skills are not required to start practicing, the long duration of rallies increases player enjoyment, it can be played outdoors, and its equipment is inexpensive [2,5,6]. Because padel is played largely in recreational environments [3], the sport seems to play an important role in promoting physical habits.

Investigations in padel have increased in the last few years [7], and have mainly focused on describing match activity and detecting effective performance indicators [8] in four fundamental aspects: temporal structure [9,10,11], players’ movements and distance covered on the court [12,13,14], design and validation of observation instruments [15,16], and game actions, such as technical or tactical parameters [8,17,18,19]. These researchers have sought to identify performance indicators that describe and explain effective players’ behaviours during the competition [11] with the aim to provide objective information on real game situations [20]. For example, occupying offensive positions close to the net seems to be a determinant to winning the point in padel [14,21]. These investigations have shown that more than 80% of the padel winning points are completed from the attack position, using different strokes such as volleys (20–25%), the tray, and the smash (12–18%) [12,22,23].

However, these results may be especially relevant when analysed according to the result of the match, since they would show the strokes that are most used to win a padel match. The results of some studies indicated that the winning pairs perform a significantly higher percentage of smashes and volleys and a lower number of ground strokes, walls strokes, and lobs than the losing pairs [24]. Moreover, padel players’ performance was characterised by the ratio between winning shots and errors [25]. Considering the effectiveness of the strokes, the winning pairs achieved a higher percentage of winners (5.6%) and a lower percentage of errors (7.5%) than the losing pairs [18] in areas close to the net [23,26]. However, studies about winners and error distribution in failed to distinguish between the different padel strokes [26]. 

Previous researchers have found sex-related differences during competition [22,27,28]. Higher values in play time, total time, as well as in the number and type of strokes have been observed in women than in men [27]. Hence, different performance profiles of padel could exist respective of sex [28]. However, information about performance indicators regarding sex or tournament round is still limited. This information is vital for planning specific and effective training sessions [6], designing players’ tactics for better performance, and developing sport injury prevention programs [18]. Therefore, the aim of this study was to analyse different performance stroke indicators in a large sample of professional padel matches (four World Padel Tour seasons) and their influence on match outcome with regard to sex, tournament round, and set number.

## 2. Materials and Methods

### 2.1. Sample and Variables

The sample contained 1070 sets from 532 matches corresponding to the tournaments in which World Padel Tour provided statistics during the 2016 to 2019 seasons. Sets were classified according to tournament round and sex: quarter finals (men: *n* = 375; women: *n* = 171), semi-finals (men: *n* = 174; women: *n* = 172), and finals (men: *n* = 83; women: *n* = 95). Sets decided with a tie-break (7-6) were excluded. The matches were played following the official game regulations [2]. The ethics board of the local university reviewed and approved the study. The following variables were analysed: sex (male and female matches), round (quarter-finals, semi-finals, and finals), game result (winning and losing pair), stroke effectiveness (smash winners, volley winners, total number of winners, and total number of errors), number of break points and break points won.

- Sex: male and female matches.
- Tournament round: quarter-finals, semi-finals, and finals.
- Set number: first set, second set, and third set.
- Match outcome: Winning and losing pair.
- Performance variables (*N*):
  ○ Stroke effectiveness: total smashes, smash winners, volley winners, total winners, and total errors.
  ○ Break points: break points and break points won.

### 2.2. Procedure

The matches were downloaded from the official channel of the World Padel Tour (https://www.youtube.com/user/WorldPadelTourAJPP (accessed on 1 July 2020). Supplementary Material Table S1 shows the links to each video. Lince video analysis software (Observesport, 1.0, Barcelona, Spain) was used to collect and register the data [29]. One observer, specialising in padel (over 5 years’ experience), was specifically trained to perform the analysis of the recordings. The observer was specifically trained in the use of the observational instrument over two weeks. The training focused on the clear identification of the performance variables (smash winners, volley winners, total number of winners, and total number of errors). Once the observer finished the training process, they analysed a total of 107 sets to calculate inter-rater reliability. These results were compared with the official match statistics. Consistency of records was analysed using the free-marginal multirater kappa [30] and the weighted kappa [31]. The score obtained was *k* = 0.93, indicating a very good strength of agreement with scores over 0.92 [32].

### 2.3. Data Analysis

Performance statistics of the matches were entered onto a spreadsheet (Microsoft Excel, Redmon, USA) for processing purposes. From the spreadsheet, the data were exported to the IBM SPSS 25.0 statistical package for Macintosh (IBM Corp: Armonk, NY, USA) for analysis. Performance variables were categorised game-by-game [33]. Then, a descriptive exploration of the data obtained was carried out and mean (*M*) and standard deviation (*SD*) were calculated. Subsequently, the Kolmogorov–Smirnov tests were performed for the study of normality and the Levene test for the homogeneity of variances. We compared the statistics on the performance variables according to match outcome, sex, and tournament round using the Mann–Whitney U-test. The effect size was calculated from Cohen’s *d* [34]. The Cohen’s *d* effect size was interpreted as small (0.20–0.50), medium (0.50–0.80), or large (>0.80) [35]. A significance level of *p* < 0.05 was established.

## 3. Results

Table 1 shows performance differences between winning and losing players according to players’ sex. Winning pairs showed significantly higher values in break points won, break points, smash winners, smashes, volley winners, and total winners, and significantly lower values in errors in both men and women. Furthermore, the effect size was large in break points won, break points, winners, and volley winners for both male and female players. 

Table 2 shows performance differences between winning and losing players according to players’ sex and tournament round. All performance variables, except smashes in the semi-final round, showed statistical differences between winning and losing pairs in the three rounds analysed for both male and female players. Furthermore, these differences decreased in the most of the performance variables from quarter-finals to finals, especially in the female category. However, the effect size in male smash winners and female volley winners increased from the quarter-finals to the finals.

Table 3 shows performance differences between winning and losing players according to player sex and set number. All performance variables, except smashes in the second and third sets and female volley winners in the third set, showed statistical differences between winning and losing pairs in the three sets analysed for both male and female players. Furthermore, differences in break points won, break points, and winners decreased during the match because the effect size is lower from the first to third sets, for both the male and female categories. Effect size also decreased from the first to third sets in the female category for volley and smash winners. However, the effect size increased from the first to third sets in errors for male and female players, and also in male volley and smash winners.

## 4. Discussion

The aim of this study was to analyse different performance stroke indicators in a large sample of matches (four World Padel Tour seasons) and their influence on match outcomes regarding sex, tournament round, and set number. This study highlighted the differences between winners and losers in some performance indicators for players. Several investigations have analysed these parameters, but mainly in the male category and not in a large sample of matches and tournaments [23,36]. This is also the first study to classify these performance variables according to tournament round and set number. The main results showed that the winning pairs demonstrated a significantly higher number of break points won, break points, smash winners, smashes, winners, and volley winners, and a significantly lower number of errors in both men and women; these results are similar to those of other studies that analysed the strokes that are most used to win a padel match [23]. Considering that smash and volley shots are performed in offensive positions, these results confirm the data already provided by similar studies, which suggest that winning players perform a significantly higher percentage of shots in positions close to the net [8,18]. More specifically, winning players performed more attack strokes in 85% of the points in a padel match [37], and the winning shots were performed with flat and topspin smashes [25].

One of the main contributions of this study is that winning pairs showed a significantly higher number of break points played and break points won, confirming that padel players must be effective when they are returning and trying to move to the offensive position during the first shots of the rally [18]. An investigation of padel has illustrated the advantage of serving by comparing points won by servers and receiving players after different numbers of shots within rallies, which lasted until shot 7 in women and shot 12 in men [19]. Therefore, coaches should consider designing exercises during training seasons including the return of serves to the server and defensive shots, such as deep lobs to the corners of the court [7,38,39], as well as strategies to win the point when players are not serving [40]. Interestingly, our findings showed a significantly lower number of errors for winning pairs, in both the male and female categories. Similar results were reported by previous studies that quantified the number of errors in professional male players, indicating that 40% of the errors are made during the first 4 s of the rally [6]. Decision-making might account for these differences by a winning players’ shot selection when hitting the ball, varying directions, and height and enhancing scoring options [8,25]. Considering these results, it seems that an effective game style when players are in the return-of-serve situation will increase the chance of executing more break points and less errors, so the possibilities to win the padel match in male and female categories would rise.

In regard to the tournament round and set number, all performance variables, except smashes, showed statistical differences between winning and loser pairs in the three rounds and sets analysed, for both male and female players. However, the differences in stroke effectiveness and break point variables between winning and losing pairs decreased during the tournament (from the quarter-finals to the finals), especially in the female category, and during the match (from the first to third set). These data confirm that there is a significantly increased equality of the scores in the last rounds of the tournament, and when the players play a third set in a match. This is remarkable considering that 30% of the matches in professional padel are decided in a third set [41]. Thus, the results of this investigation imply special attention should be paid to conditioning sessions to incorporate decider sets and matches to better replicate game-like conditions, as it is well-understood that high cardiorespiratory capacity and muscular endurance in players delays fatigue and aids in recovery [42].

The current study adds novel insights into notational analysis in padel, considering, for the first time, a large sample of tournaments and seasons. However, some limitations to the study should be noted. First, the number of performance indicators analysed is low. Future research should consider studying other variables such as court zones, shot directions, or players’ hand-dominance and game side, due to their relation to padel match outcomes [8,24,25]. Second, we analysed only three tournament rounds; future studies should consider including qualifying rounds. Finally, although these data constitute a useful guide for training designs and injury prevention programs, notational analysis studies should explore game dynamics in padel through shot-by-shot analysis.

## 5. Conclusions

This study presented new contributions on performance indicators in professional padel. Shot effectiveness seemed to be a key factor in professional padel that distinguished between winning and losing players. The data showed that winning pairs demonstrated a significantly higher number of break points won, total break points, smash winners, total smashes, total winners, volley winners, and a significantly lower number of errors, in both men and women. Moreover, the match equality increased during the tournament (from the quarter-finals to the finals), and during the match (from the first to third set). This knowledge may have implications in the design of appropriate game strategies and specific training sessions to improve performance and to prevent sport injuries. The findings of this study suggest that coaches should consider training volleys and smash winners, returns of serve, and defensive shots such as lobs to enhance winning options. As a practical application, sex differences should be considered when coaches prescribe sessions based on stroke volume, due to women performing more volleys and men more smashes.

## Figures and Tables

**Table 1:** Performance differences between winning and losing players according to players’ sex.

Performance Variables (*N*)	Men					Women				
	Winning Pair	Loser Pair				Winning Pair	Losing Pair	Total		
	*M*	*M*	*U*	*p*	*d*	*M*	*M*	*U*	*p*	*d*
Break points won	0.22	0.04	31,409.50	0.00 *	2.13	0.26	0.06	13,543.50	0.00 *	2.22
Break points	0.46	0.20	84,491.50	0.00 *	1.15	0.55	0.28	43,021.00	0.00 *	1.08
Errors	0.77	1.04	117,438.00	0.00 *	0.76	0.94	1.21	61,342.00	0.00 *	0.65
Smash winners	1.01	0.73	108,996.00	0.00 *	0.85	0.67	0.44	59,784.50	0.00 *	0.69
Smashes	1.56	1.40	35,024.00	0.00 *	0.29	1.07	0.91	23,729.00	0.00 *	0.27
Winners	1.51	1.09	20,040.50	0.00 *	1.01	1.75	1.21	11,512.00	0.00 *	1.18
Volley winners	0.91	0.60	26,356.50	0.00 *	1.08	0.96	0.65	10,472.00	0.00 *	0.91

Note: *M* = Mean; * = *p* < 0.05: *d* = effect size.

**Table 2:** Performance differences between winning and losing players according to players’ sex and tournament round.

Performance Variables (*N*)	Men											
	Quarter-Finals				Semi-Finals				Finals			
	Winning Pair	Losing Pair			Winning Pair	Losing Pair			Winning Pair	Losing Pair		
	*M*	*M*	*p*	*d*	*M*	*M*	*p*	*d*	*M*	*M*	*p*	*d*
Break points won	0.22	0.05	0.00 *	2.10	0.22	0.05	0.00 *	2.29	0.21	0.04	0.00 *	1.97
Break points	0.47	0.29	0.00 *	1.24	0.45	0.22	0.00 *	1.00	0.47	0.20	0.00 *	1.07
Errors	0.77	1.04	0.00 *	0.80	0.79	1.03	0.00 *	0.67	0.76	1.05	0.00 *	0.77
Smash winners	1.00	0.73	0.00 *	0.80	1.01	0.72	0.00 *	0.91	1.07	0.75	0.00 *	0.95
Smashes	1.57	1.40	0.00 *	0.30	1.46	1.39	0.24	0.18	1.72	1.45	0.04 *	0.45
Winners	1.50	1.08	0.00 *	1.11	1.52	1.11	0.00 *	1.02	1.50	1.14	0.00 *	0.67
Volley winners	0.92	0.60	0.00 *	1.08	0.91	0.58	0.00 *	1.16	0.83	0.62	0.00 *	0.83
**Performance Variables** **(*N*)**	**Women**											
	**Quarter-Finals**				**Semi-Finals**				**Finals**			
	**Winning Pair**	**Losing Pair**			**Winning Pair**	**Losing Pair**			**Winning Pair**	**Losing Pair**		
	***M***	***M***	***p***	***d***	***M***	***M***	***p***	***d***	***M***	***M***	***p***	***d***
Break points won	0.28	0.07	0.00 *	2.32	0.25	0.06	0.00 *	2.18	0.26	0.07	0.00 *	2.10
Break points	0.56	0.25	0.00 *	1.35	0.54	0.29	0.00 *	0.97	0.53	0.31	0.00 *	0.84
Errors	0.92	1.23	0.00 *	0.78	0.95	1.19	0.00 *	0.56	0.96	1.22	0.00 *	0.60
Smash winners	0.63	0.39	0.00 *	0.78	0.70	0.47	0.00 *	0.64	0.68	0.46	0.00 *	0.63
Smashes	1.03	0.85	0.04 *	0.31	1.06	0.98	0.43	0.11	1.18	0.90	0.01 *	0.50
Winners	1.78	1.16	0.00 *	1.33	1.69	1.19	0.00 *	1.15	1.79	1.32	0.00 *	0.93
Volley winners	0.96	0.67	0.00 *	0.90	0.96	0.65	0.00 *	0.86	0.95	0.62	0.00 *	0.94

Note: *M* = Mean; * = *p* < 0.05: *d* = effect size.

**Table 3:** Performance differences between winning and losing players according to players’ sex and set number.

Performance Variables (*N*)	Men											
	First Set				Second Set				Third Set			
	Winning Pair	Losing Pair			Winning Pair	Losing Pair			Winning Pair	Losing Pair		
	*M*	*M*	*p*	*d*	*M*	*M*	*p*	*d*	*M*	*M*	*p*	*d*
Break points won	0.23	0.04	0.00 *	2.18	0.22	0.05	0.00 *	2.11	0.20	0.05	0.00 *	2.02
Break points	0.47	0.19	0.00 *	1.23	0.46	0.21	0.00 *	1.11	0.43	0.21	0.00 *	0.98
Errors	0.78	1.04	0.00 *	0.76	0.77	1.04	0.00 *	0.73	0.74	1.03	0.00 *	0.84
Smash winners	0.99	0.69	0.00 *	0.90	1.03	0.77	0.00 *	0.78	1.02	0.73	0.00 *	0.91
Smashes	1.53	1.32	0.00 *	0.40	1.62	1.51	0.17	0.17	1.46	1.31	0.13	0.35
Winners	1.52	1.06	0.00 *	1.14	1.50	1.10	0.00 *	0.95	1.48	1.18	0.00 *	0.81
Volley winners	0.90	0.58	0.00 *	1.10	0.92	0.62	0.00 *	1.02	0.89	0.55	0.00 *	1.14
**Performance Variables** **(*N*)**	**Women**											
	**First Set**				**Second Set**				**Third Set**			
	**Winning Pair**	**Losing Pair**			**Winning Pair**	**Losing Pair**			**Winning Pair**	**Losing Pair**		
	***M***	***M***	***p***	***d***	***M***	***M***	***p***	***d***	***M***	***M***	***p***	***d***
Break points won	0.26	0.06	0.00 *	2.26	0.27	0.07	0.00 *	2.18	0.26	0.06	0.00 *	2.10
Break points	0.54	0.27	0.00 *	1.08	0.54	0.27	0.00 *	0.97	0.76	0.32	0.00 *	0.84
Errors	0.97	1.24	0.00 *	0.66	0.92	1.18	0.00 *	0.56	0.88	1.23	0.00 *	0.60
Smash winners	0.66	0.41	0.00 *	0.75	0.67	0.46	0.00 *	0.64	0.68	0.48	0.00 *	0.63
Smashes	1.06	0.84	0.00 *	0.41	1.06	0.97	0.40	0.11	1.15	0.93	0.27	0.50
Winners	1.69	1.17	0.00 *	1.14	1.81	1.21	0.00 *	1.15	1.74	1.32	0.00 *	0.93
Volley winners	1.02	0.62	0.00 *	1.26	0.91	0.68	0.00 *	0.86	0.88	0.67	0.09	0.94

Note: *M* = Mean; * = *p* < 0.05: *d* = effect size.

## Supplementary Materials

The following are available online at https://www.mdpi.com/article/10.3390/ijerph18094904/s1. Table S1: links to each video.
