# Supplementary material for: Analysis of Game Performance Indicators during 2015–2019 World Padel Tour Seasons and Their Influence on Match Outcome
Source: Int J Environ Res Public Health. 2021 May 4;18(9):4904. doi: 10.3390/ijerph18094904 (PMC8125176; doi:10.3390/ijerph18094904)
Supplement: Supplementary file 1 [file ijerph-18-04904-s001.zip › Table S1, links to each video-2.docx]

| Season | Tournament | Number of Matches per phase  (Nº Male + Nº Female) | Number of Sets  (Nº Male + Nº Female) | Links |
| --- | --- | --- | --- | --- |
| 2016 | WPT Valencia Master | 6 Quarter Finals (4M+2F) | 12 (8M+4F) | https://www.youtube.com/watch?v=c38xiUr2EOk&t=12022s  https://www.youtube.com/watch?v=4RYoJ7FcmVw&t=14178s |
|  |  | 4 Semi Finals (2M+2F) | 8 (4M+4F) | https://www.youtube.com/watch?v=9wC7B19eJ7Q  https://www.youtube.com/watch?v=MwZz24EQr0g |
|  |  | 1 Final (1F) | 1 (1F) | https://www.youtube.com/watch?v=OsThkyCLfR8&t=8356s |
|  | WPT Barcelona Master | 6 Quarter Finals (4M+2F) | 11 (7M+4F) | https://www.youtube.com/watch?v=R7M_zUn19DU&t=3276s |
|  |  | 3 Semi Finals (2M+1F) | 7 (5M+2F) | https://www.youtube.com/watch?v=xEo1R16PeyA  https://www.youtube.com/watch?v=97TZsF5dTyA |
|  |  | 2 Finals (1M+1F) | 5 (2M+3F) | https://www.youtube.com/watch?v=qmLMIaFP9uY&t=9462s  https://www.youtube.com/watch?v=IE_kx7Z1_ig |
|  | WPT Las Rozas Open | 4 Quarter Finals (4M) | 8 (8M) | https://www.youtube.com/watch?v=0RkuhnqxbzY |
|  |  | 4 Semi Finals (2M+2F) | 8 (3M+5F) | https://www.youtube.com/watch?v=s6sfQtgV0ew&t=14798s  https://www.youtube.com/watch?v=Q0aCrA8zalc |
|  |  | 2 Finals (1M+1F) | 5 (2M+3F) | https://www.youtube.com/watch?v=hDB88zGxjyA  https://www.youtube.com/watch?v=-3iP54IcAYE&t=5773s |
|  | WPT Mallorca Open | 4 Quarter Finals (4M) | 9 (9M) | https://www.youtube.com/watch?v=clX1Eg5M2h0&t=4745s |
|  |  | 2 Semi Finals (2F) | 4 (4F) | https://www.youtube.com/watch?v=MFV3ea69W9I |
|  |  | 1 Final (1F) | 3 (3F) | https://www.youtube.com/watch?v=j-g52b_Ximk |
|  | WPT Valladolid Open | 4 Quarter Finals (4M) | 8 (8M) | https://www.youtube.com/watch?v=hmlj3_s7nG8&t=27767s |
|  |  | 4 Semi Finals (2M+2F) | 9 (5M+4F) | https://www.youtube.com/watch?v=OobKY2O-wdo  https://www.youtube.com/watch?v=S0AWq175seQ&t=15050s |
|  |  | 2 Finals (1M+1F) | 3 (1M+2F) | https://www.youtube.com/watch?v=ZrPI4qFj1Cc&t=3868s  https://www.youtube.com/watch?v=Vka2YyFl030&t=8042s |
|  | WPT Gran Canaria Open | 4 Quarter Finals (4M) | 8 (8M) | https://www.youtube.com/watch?v=B8vF1_ohWEw |
|  |  | 2 Semi Finals (2M) | 4 (4M) | https://www.youtube.com/watch?v=EDtniFF_Rgs |
|  |  | 1 Final (1M) | 2 (2M) | https://www.youtube.com/watch?v=GZXYbGN9OFg&t=6144s |
|  | WPT La nucia Open | 4 Quarter Finals (4M) | 9 (9M) | https://www.youtube.com/watch?v=xrzlb0or4lc |
|  |  | 2 Semi Finals (2M) | 3 (3M) | https://www.youtube.com/watch?v=YJzLcii9ZJU |
|  |  | 1 Final (1F) | 1 (1F) | https://www.youtube.com/watch?v=JTcWIOV5uKg&t=9064s |
|  | WPT Monte-carlo Master | 4 Quarter Finals (4M) | 8 (8M) | https://www.youtube.com/watch?v=_j1x0tZ76KU&t=26874s |
|  |  | 4 Semi Finals (2M+2F) | 7 (3M+4F) | https://www.youtube.com/watch?v=TtRjGCL89bE  https://www.youtube.com/watch?v=ORxIYoh_cb4 |
|  |  | 2 Finals (1M+1F) | 4 (2M+2F) | https://www.youtube.com/watch?v=Btn_eqqYMKM  https://www.youtube.com/watch?v=LG2wfyagVZw&t=6319s |
|  | WPT Sevilla Open | 6 Quarter Finals (4M+2F) | 10 (7M+3F) | https://www.youtube.com/watch?v=ggxCYndjegg  https://www.youtube.com/watch?v=5FeWl_slor8 |
|  |  | 4 Semi Finals (2M+2F) | 7 (4M+3F) | https://www.youtube.com/watch?v=-cdp9Zo51bE  https://www.youtube.com/watch?v=BCSMQ8_Y6Uk |
|  |  | 2 Finals (1M+1F) | 4 (2M+2F) | https://www.youtube.com/watch?v=S1O2GE1m_iQ&t=6589s  https://www.youtube.com/watch?v=ngBd7KlQbR8&t=5674s |
|  | WPT A Coruña Open | 6 Quarter Finals (4M+2F) | 14 (9M+5F) | https://www.youtube.com/watch?v=eF8_Q3vIXP0&t=17166s  https://www.youtube.com/watch?v=GKCp4np_6_o |
|  |  | 4 Semi Finals (2M+2F) | 8 (4M+4F) | https://www.youtube.com/watch?v=ynAIj0s09wE  https://www.youtube.com/watch?v=oPtkkk53Vv0 |
|  |  | 2 Finals (1M+1F) | 4 (2M+2F) | https://www.youtube.com/watch?v=t-cMeIdqkAI&t=11314s  https://www.youtube.com/watch?v=1muumO2Yp6A&t=8618s |
|  | WPT Zaragoza Open | 6 Quarter Finals (4M+2F) | 13 (8M+5F) | https://www.youtube.com/watch?v=UUcjigMo7Yg  https://www.youtube.com/watch?v=SfDl0KmMxvM |
|  |  | 4 Semi Finals (2M+2F) | 7 (4M+3F) | https://www.youtube.com/watch?v=Q8_nD59ZFkE  https://www.youtube.com/watch?v=60jeXRiYvZU |
|  |  | 2 Finals (1M+1F) | 4 (2M+2F) | https://www.youtube.com/watch?v=WVsdzlP6bJo&t=7427s  https://www.youtube.com/watch?v=0OblAALesCY&t=4858s |
|  | WPT Euskadi Open | 5 Quarter Finals (3M+2F) | 10 (5M+5F) | https://www.youtube.com/watch?v=AS6LLZhNUCI&t=2332s  https://www.youtube.com/watch?v=XTLhY78A1gw |
|  |  | 4 Semi Finals (2M+2F) | 9 (5M+4F) | https://www.youtube.com/watch?v=4B6cy1OwyVY  https://www.youtube.com/watch?v=XQX2iS2OCgg |
|  |  | 2 Finals (1M+1F) | 4 (2M+2F) | https://www.youtube.com/watch?v=iI-tjbqK040&t=7706s  https://www.youtube.com/watch?v=Qrhb27qTxoo&t=6153s |
|  | WPT Master Final Madrid | 5 Quarter Finals (3M+2F) | 9 (5M+4F) | https://www.youtube.com/watch?v=dx4KrDqI7pk  https://www.youtube.com/watch?v=IDVKNk_uiiU&t=11071s |
|  |  | 3 Semi Finals (1M+2F) | 5 (2M+3F) | https://www.youtube.com/watch?v=IG6H58oN1KA&t=2501s  https://www.youtube.com/watch?v=rWvkwBET5fA |
|  |  | 2 Finals (1M+1F) | 3 (2M+1F) | https://www.youtube.com/watch?v=SyWaBvW07JE  https://www.youtube.com/watch?v=-nPg0dVFPxs |
| 2017 | WPT Santander Open | 6 Quarter Finals (4M+2F) | 12 (8M+4F) | https://www.youtube.com/watch?v=Yd_w5BCJ3Z4  https://www.youtube.com/watch?v=X_GvZzBaitI |
|  |  | 4 Semi Finals (2M+2F) | 8 (4M+4F) | https://www.youtube.com/watch?v=fLNH5jNEIA4  https://www.youtube.com/watch?v=QPvcbRvjKQg |
|  |  | 2 Finals (1M+1F) | 4 (3M+1F) | https://www.youtube.com/watch?v=6RoE7Tn78w0 |
|  | WPT A Coruña Open | 6 Quarter Finals (4M+2F) | 13 (10M+3F) | https://www.youtube.com/watch?v=kOCfskJk5A4&t=19s  https://www.youtube.com/watch?v=RR6JsqE3YP4&t=9s |
|  |  | 4 Semi Finals (2M+2F) | 8 (4M+4F) | https://www.youtube.com/watch?v=ghT455aW5j0  https://www.youtube.com/watch?v=_Pt-4xlJL94&t=11s |
|  | WPT Barcelona master | 5 Quarter Finals (3M+2F) | 10 (7M+3F) | https://www.youtube.com/watch?v=3EGKDtR3__E |
|  |  | 2 Semi Finals (1M+1F) | 3 (1M+2F) | https://www.youtube.com/watch?v=00c9cDq8H7c |
|  |  | 2 Finals (1M+1F) | 5 (2M+3F) | https://www.youtube.com/watch?v=MKxmCbh-_-8  https://www.youtube.com/watch?v=ypqZKh8AZoo |
|  | WPT Valladolid Open | 6 Quarter Finals (4M+2F) | 14 (10M+4F) | https://www.youtube.com/watch?v=d8BL3iiniZw  https://www.youtube.com/watch?v=cj1jvwPRvvk |
|  |  | 4 Semi Finals (2M+2F) | 7 (3M+4F) | https://www.youtube.com/watch?v=N7RApRuI4mw  https://www.youtube.com/watch?v=GINdKV2AZVY  https://www.youtube.com/watch?v=ZR-yJiRs1BQ  https://www.youtube.com/watch?v=VRfKzT54dkw |
|  | WPT Mijas Open | 6 Quarter Finals (4M+2F) | 12 (7M+5F) | https://www.youtube.com/watch?v=Hey5heciofA  https://www.youtube.com/watch?v=QPDttoaToAk |
|  |  | 4 Semi Finals (2M+2F) | 9 (5M+4F) | https://www.youtube.com/watch?v=jnEsu57dauk  https://www.youtube.com/watch?v=AmfCsx7vM2w  https://www.youtube.com/watch?v=HsIvYAttGts  https://www.youtube.com/watch?v=iZXCAPPvdxA |
|  |  | 2 Finals (1M+1F) | 4 (3M+1F) | https://www.youtube.com/watch?v=anlfTEG0sHM&t=3636s  https://www.youtube.com/watch?v=sDgpvNNLOHI |
|  | WPT Gran Canaria Open | 4 Quarter Finals (4M) | 6 (6M) | https://www.youtube.com/watch?v=6qvGwNRaoQ0 |
|  |  | 1 Semi Finals (1M) | 1 (1M) | https://www.youtube.com/watch?v=m2WwAJ8DU0E |
|  |  | 1 Final (1M) | 2 (2M) | https://www.youtube.com/watch?v=O8Q3xAlEZPs |
|  | WPT Alicante Open | 6 Quarter Finals (4M+2F) | 13 (9M+4F) | https://www.youtube.com/watch?v=AozIo_IPvrE  https://www.youtube.com/watch?v=k_CIB2vTKdg |
|  |  | 4 Semi Finals (2M+2F) | 9 (5M+4F) | https://www.youtube.com/watch?v=3-PQkuIG1GU  https://www.youtube.com/watch?v=LDHvU2pelBA |
|  |  | 2 Final (1M+1F) | 4 (3M+1F) | https://www.youtube.com/watch?v=fVycCNvgFJ8 |
|  | WPT Sevilla Open | 6 Quarter Finals (4M+2F) | 13 (8M+5F) | https://www.youtube.com/watch?v=_jtbSEYs6-k  https://www.youtube.com/watch?v=xhAS0JMgba8 |
|  |  | 4 Semi Finals (2M+2F) | 6 (3M+3F) | https://www.youtube.com/watch?v=Fwkn45aYZQU  https://www.youtube.com/watch?v=1DEZ95h99vA |
|  |  | 2 Final (1M+1F) | 4 (2M+2F) | https://www.youtube.com/watch?v=Wc9ZZMdtD7w |
|  | WPT Andorra Open | 4 Quarter Finals (4M) | 7 (7M) | https://www.youtube.com/watch?v=ESy5vdrq43E |
|  |  | 2 Semi Finals (2M) | 6 (6M) | https://www.youtube.com/watch?v=V8YTFjxCIu8  https://www.youtube.com/watch?v=M8chVj4nD_A |
|  |  | 1 Final (1F) | 2 (2F) | https://www.youtube.com/watch?v=V8YTFjxCIu8 |
|  | WPT Granada Open | 6 Quarter Finals (4M+2F) | 13 (9M+4F) | https://www.youtube.com/watch?v=d2HcEN663pM  https://www.youtube.com/watch?v=FOwChpryRBg |
|  |  | 4 Semi Finals (2M+2F) | 8 (5M+3F) | https://www.youtube.com/watch?v=Fbml7OJc__Q  https://www.youtube.com/watch?v=LZ_DT8LDf_E |
|  |  | 2 Finals (1M+1F) | 3 (1M+2F) | https://www.youtube.com/watch?v=nlrU6cjs5l8 |
|  | WPT Zaragoza Open | 5 Quarter Finals (3M+2F) | 10 (6M+4F) | https://www.youtube.com/watch?v=u1wN1bOJIIs  https://www.youtube.com/watch?v=ffPKws8Shis |
|  |  | 4 Semi Finals (2M+2F) | 9 (5M+4F) | https://www.youtube.com/watch?v=mmPPx94iWQ0&t=13057s  https://www.youtube.com/watch?v=GTDBn_U5deA |
|  |  | 2 Finals (1M+1F) | 5 (2M+3F) | https://www.youtube.com/watch?v=1sUa--gttHM |
|  | WPT Keler Bilbao Open | 6 Quarter Finals (4M+2F) | 13 (9M+4F) | https://www.youtube.com/watch?v=Ti2rIiG6NY8  https://www.youtube.com/watch?v=_D0wO5NyI3k |
|  |  | 4 Semi Finals (2M+2F) | 6 (4M+2F) | https://www.youtube.com/watch?v=XDtyoeq_uKc  https://www.youtube.com/watch?v=qdQV3REGTPk |
|  |  | 2 Finals (1M+1F) | 4 (2M+2F) | https://www.youtube.com/watch?v=m6LNXgoqwKw&t=15590s |
|  | WPT Master Final | 8 Quarter Finals (4M+4F) | 20 (10M+10F) | https://www.youtube.com/watch?v=9MuvFaXSw5U  https://www.youtube.com/watch?v=xBEDy-ions0 |
|  |  | 4 Semi Finals (2M+2F) | 8 (4M+4F) | https://www.youtube.com/watch?v=FCBnI6gek8M  https://www.youtube.com/watch?v=SpFd0US9EHc |
|  |  | 2 Finals (1M+1F) | 4 (2M+2F) | https://www.youtube.com/watch?v=SKiqZ43k9wA&t=13034s |
| 2018 | WPT Zaragoza Open | 5 Quarter Finals (4M+1F) | 12 (10M+2F) | https://www.youtube.com/watch?v=YxFtlWQIPvI  https://www.youtube.com/watch?v=0Pm3_9hfqxY&t=2321s |
|  |  | 3 Semi Finals (1M+2F) | 8 (2M+6F) | https://www.youtube.com/watch?v=JHwkLCDBgAQ&t=10190s  https://www.youtube.com/watch?v=aPv0gAmMIlM&t=12909s |
|  |  | 2 Finals (1M+1F) | 4 (2M+2F) | https://www.youtube.com/watch?v=W3WOgBRs-VA |
|  | WPT Jaen Open | 6 Quarter Finals (4M+2F) | 13 (9M+4F) | https://www.youtube.com/watch?v=jGmq4aoU0rY  https://www.youtube.com/watch?v=KVl48_1SU64 |
|  |  | 3 Semi Finals (1M+2F) | 5 (1M+4F) | https://www.youtube.com/watch?v=UwyYaCY3yjM  https://www.youtube.com/watch?v=vegiY1mGm2s |
|  |  | 2 Finals (1M+1F) | 4 (2M+2F) | https://www.youtube.com/watch?v=d-vC_pIJLoU |
|  | WPT Valladolid Open | 6 Quarter Finals (4M+2F) | 10 (6M+4F) | https://www.youtube.com/watch?v=97J6Nj4AGnU  https://www.youtube.com/watch?v=T46c3ilAH1E&t=39318s |
|  |  | 4 Semi Finals (2M+2F) | 7 (3M+4F) | https://www.youtube.com/watch?v=5y9pqbY67Z0  https://www.youtube.com/watch?v=5hrrhGXIdoQ |
|  |  | 2 Finals (1M+1F) | 6 (3M+3F) | https://www.youtube.com/watch?v=gUO06leaNBQ |
|  | WPT Valencia Master | 5 Quarter Finals (3M+2F) | 10 (6M+4F) | https://www.youtube.com/watch?v=n97kEUwlfps  https://www.youtube.com/watch?v=FK5Qd4S_r8I |
|  |  | 4 Semi Finals (2M+2F) | 9 (5M+4F) | https://www.youtube.com/watch?v=p2FevN7ArM8  https://www.youtube.com/watch?v=vVP34M90G8c |
|  |  | 2 Finals (1M+1F) | 4 (2M+2F) | https://www.youtube.com/watch?v=DdtJc2C9xbg |
|  | WPT Mijas Open | 4 Quarter Finals (3M+1F) | 7 (5M+2F) | https://www.youtube.com/watch?v=H0LFfDaxh2E&t=9319s  https://www.youtube.com/watch?v=YQuWsgD0r78 |
|  |  | 4 Semi Finals (2M+2F) | 5 (2M+3F) | https://www.youtube.com/watch?v=FkJHyCOrxo0  https://www.youtube.com/watch?v=ud59l0G87cE |
|  |  | 2 Finals (1M+1F) | 4 (2M+2F) | https://www.youtube.com/watch?v=pyzPoWSyRyA&t=3557s |
|  | WPT Andorra Open | 6 Quarter Finals (4M+2F) | 10 (6M+4F) | https://www.youtube.com/watch?v=BSQGjdCN4RQ  https://www.youtube.com/watch?v=PZ3xte6gWYA |
|  |  | 4 Semi Finals (2M+2F) | 8 (4M+4F) | https://www.youtube.com/watch?v=PDZjiBrNzb8  https://www.youtube.com/watch?v=JVf5gm_VERM |
|  |  | 2 Finals (1M+1F) | 4 (3M+1F) | https://www.youtube.com/watch?v=A7vhS56cBs4&t=19422s |
|  | WPT Lugo Open | 6 Quarter Finals (4M+2F) | 11 (8M+3F) | https://www.youtube.com/watch?v=vdANHmukYvA  https://www.youtube.com/watch?v=hLROJPyeqL0 |
|  |  | 3 Semi Finals (1M+2F) | 5 (1M+4F) | https://www.youtube.com/watch?v=ABPU0ngy_AQ  https://www.youtube.com/watch?v=Ie6nJwwoVtA |
|  |  | 2 Finals (1M+1F) | 4 (2M+2F) | https://www.youtube.com/watch?v=zoYhi4_xSRA |
|  | WPT Granada Open | 6 Quarter Finals (4M+2F) | 11 (7M+4F) | https://www.youtube.com/watch?v=Y0sHtlm3Hfk  https://www.youtube.com/watch?v=ZZmDfSEBIdY |
|  |  | 4 Semi Finals (2M+2F) | 8 (4M+4F) | https://www.youtube.com/watch?v=W4uTRmTwCNo  https://www.youtube.com/watch?v=MN7zZkws2f0 |
|  |  | 2 Finals (1M+1F) | 3 (1M+2F) | https://www.youtube.com/watch?v=fmsDg_cRIkw |
|  | WPT Bilbao Open | 6 Quarter Finals (4M+2F) | 12 (8M+4F) | https://www.youtube.com/watch?v=Bx8sg8KmB7g  https://www.youtube.com/watch?v=nMps3X8qlM0 |
|  |  | 4 Semi Finals (2M+2F) | 8 (4M+4F) | https://www.youtube.com/watch?v=ybaZJKVzp10  https://www.youtube.com/watch?v=hxm4p4rzo1Y |
|  |  | 2 Finals (1M+1F) | 5 (2M+3F) | https://www.youtube.com/watch?v=wu3Tzx3xsfU |
|  | WPT Murcia Open | 5 Quarter Finals (3M+2F) | 10 (7M+3F) | https://www.youtube.com/watch?v=lUK9RnqPOHA  https://www.youtube.com/watch?v=MPJw-RIJVag |
|  |  | 4 Semi Finals (2M+2F) | 7 (4M+3F) | https://www.youtube.com/watch?v=F1ZVX-6UeLg  https://www.youtube.com/watch?v=iEnfWrNmVS4  https://www.youtube.com/watch?v=kYIGz30TRBM |
|  |  | 2 Finals (1M+1F) | 6 (3M+3F) | https://www.youtube.com/watch?v=Met3C8H5Sw0 |
|  | WPT Master Final | 8 Quarter Finals (4M+4F) | 14 (7M+7F) | https://www.youtube.com/watch?v=phwYUM7UNrU  https://www.youtube.com/watch?v=-zXjg-G8UzA  https://www.youtube.com/watch?v=euG2AIoDuAQ  https://www.youtube.com/watch?v=f0m__zeqWuo |
|  |  | 4 Semi Finals (2M+2F) | 6 (2M+4F) | https://www.youtube.com/watch?v=2K6cfnP4Ee0  https://www.youtube.com/watch?v=Q6OvKNpl3LI |
|  |  | 2 Finals (1M+1F) | 3 (1M+2F) | https://www.youtube.com/watch?v=gZfPmQZ0HNk&t=14701s |
| 2019 | WPT Marbella Master | 3 Quarter Finals (1M+2F) | 7 (2M+5F) | https://www.youtube.com/watch?v=_9PR9dvGQ60&t=16319s  https://www.youtube.com/watch?v=F1aCqoIwFUw&t=11846s |
|  |  | 4 Semi Finals (2M+2F) | 8 (5M+3F) | https://www.youtube.com/watch?v=ch4BDLKBUNE  https://www.youtube.com/watch?v=zJZof_bzirs |
|  |  | 2 Finals (1M+1F) | 4 (1M+3F) | https://www.youtube.com/watch?v=dMhiMrC3vbk&t=12867s |
|  | WPT Logroño Open | 6 Quarter Finals (4M+2F) | 14 (10M+4F) | https://www.youtube.com/watch?v=89ZmHbrMocE  https://www.youtube.com/watch?v=knkOAvU891U&t=9395s |
|  |  | 4 Semi Finals (2M+2F) | 8 (3M+5F) | https://www.youtube.com/watch?v=V89fIXBG-fY  https://www.youtube.com/watch?v=0CeHIs5sb78 |
|  |  | 1 Final (1F) | 3 (3F) | https://www.youtube.com/watch?v=qo46tPoYgPc&t=19166s |
|  | WPT Alicante Open | 5 Quarter Finals (4M+1F) | 12 (9M+3F) | https://www.youtube.com/watch?v=t9QUYTG8VEk  https://www.youtube.com/watch?v=NXI1cKuhJGQ |
|  |  | 4 Semi Finals (2M+2F) | 11 (6M+5F) | https://www.youtube.com/watch?v=G--BwdUMKNE&t=1282s  https://www.youtube.com/watch?v=2f4fOldWje4&t=8447s |
|  |  | 2 Finals (1M+1F) | 4 (2M+2F) | https://www.youtube.com/watch?v=PW8MTUbTcYg&t=6825s |
|  | WPT Vigo Open | 5 Quarter Finals (3M+2F) | 10 (6M+4F) | https://www.youtube.com/watch?v=was4EG4fXLI&t=5787s  https://www.youtube.com/watch?v=a_iHLCNUC4c&t=9809s |
|  |  | 3 Semi Finals (2M+1F) | 5 (3M+2F) | https://www.youtube.com/watch?v=RpLn2gohH88&t=10414s  https://www.youtube.com/watch?v=AxNyq8S77no |
|  |  | 2 Finals (1M+1F) | 4 (2M+2F) | https://www.youtube.com/watch?v=O4k7CLvxuos&t=5487s |
|  | WPT Jaen Open | 6 Quarter Finals (4M+2F) | 11 (7M+4F) | https://www.youtube.com/watch?v=YuW613nDRzY  https://www.youtube.com/watch?v=5BRPlUK66CA |
|  |  | 4 Semi Finals (2M+2F) | 9 (3M+6F) | https://www.youtube.com/watch?v=XSRHn0QO0Kc  https://www.youtube.com/watch?v=bDlfUcA-X1Q |
|  |  | 2 Finals (1M+1F) | 5 (2M+3F) | https://www.youtube.com/watch?v=gnvK_1l-pcA |
|  | WPT Valladolid Master | 6 Quarter Finals (4M+2F) | 14 (8M+6F) | https://www.youtube.com/watch?v=Oh4mD_cgPQc  https://www.youtube.com/watch?v=ICIS1OIHWpk |
|  |  | 4 Semi Finals (2M+2F) | 7 (4M+3F) | https://www.youtube.com/watch?v=DsjzvznuoX0  https://www.youtube.com/watch?v=UlvTiT5ZRJM |
|  |  | 2 Finals (1M+1F) | 5 (2M+3F) | https://www.youtube.com/watch?v=xBU0IjwS7fI&t=18080s |
|  | WPT Valencia Open | 6 Quarter Finals (4M+2F) | 13 (8M+5F) | https://www.youtube.com/watch?v=rvnMAp09aXI  https://www.youtube.com/watch?v=AVITTcATgYs |
|  |  | 4 Semi Finals (2M+2F) | 10 (5M+5F) | https://www.youtube.com/watch?v=14rhrinq75U  https://www.youtube.com/watch?v=280bk4iRHG8 |
|  |  | 2 Finals (1M+1F) | 4 (2M+2F) | https://www.youtube.com/watch?v=w-mnb_uIe7o&t=17449s |
|  | WPT Mijas Open | 6 Quarter Finals (4M+2F) | 12 (8M+4F) | https://www.youtube.com/watch?v=4V-VD2t6_Gk  https://www.youtube.com/watch?v=f7C1gyauZdc |
|  |  | 4 Semi Finals (2M+2F) | 7 (3M+4F) | https://www.youtube.com/watch?v=fRcUggTa8g4  https://www.youtube.com/watch?v=_OPyKMmXAe0 |
|  |  | 2 Finals (1M+1F) | 3 (1M+2F) | https://www.youtube.com/watch?v=TUCW727mjNU&t=12684s |
|  | WPT Madrid Master | 6 Quarter Finals (4M+2F) | 11 (8M+3F) | https://www.youtube.com/watch?v=ksCXy6_9y2A&t=18993s  https://www.youtube.com/watch?v=U2RJBZRBNeg |
|  |  | 3 Semi Finals (1M+2F) | 5 (1M+4F) | https://www.youtube.com/watch?v=Pgky3PW5KJ8  https://www.youtube.com/watch?v=ruQ96lxyeCU&t=6946s |
|  |  | 2 Finals (1M+1F) | 4 (2M+2F) | https://www.youtube.com/watch?v=xv98LmUTtCM&t=12940s |
|  | WPT Menorca Open | 5 Quarter Finals (4M+1F) | 11 (9M+2F) | https://www.youtube.com/watch?v=-mBHziRNtA0  https://www.youtube.com/watch?v=s3J9vheFH1s |
|  |  | 4 Semi Finals (2M+2F) | 9 (4M+5F) | https://www.youtube.com/watch?v=8IjNfn86t_k  https://www.youtube.com/watch?v=NXVm7PJHn9k |
|  |  | 2 Finals (1M+1F) | 4 (2M+2F) | https://www.youtube.com/watch?v=ASCE6OX_QaM&t=18562s |
|  | WPT Cordoba Open | 6 Quarter Finals (4M+2F) | 10 (8M+2F) | https://www.youtube.com/watch?v=Ypb7Lea2yyE  https://www.youtube.com/watch?v=h8tiqJNJRVo |
|  |  | 4 Semi Finals (2M+2F) | 8 (4M+4F) | https://www.youtube.com/watch?v=UYdknaybsjI  https://www.youtube.com/watch?v=pQMl7Nih4QA |
|  |  | 2 Finals (1M+1F) | 4 (2M+2F) | https://www.youtube.com/watch?v=FtWp6BatpkI&t=11714s |
|  | WPT Master Final | 8 Quarter Finals (4M+4F) | 16 (8M+8F) | https://www.youtube.com/watch?v=JYrxzHo_7q4  https://www.youtube.com/watch?v=b4WtrxSNXwU  https://www.youtube.com/watch?v=4Kz-ybtsgT4  https://www.youtube.com/watch?v=paQgLd7Ll-A |
|  |  | 4 Semi Finals (2M+2F) | 9 (5M+4F) | https://www.youtube.com/watch?v=xIFIyqspOHo&t=9161s  https://www.youtube.com/watch?v=z_dIJFUrOds&t=15528s |
|  |  | 2 Finals (1M+1F) | 3 (1M+2F) | https://www.youtube.com/watch?v=zx_mc1Ingqg&t=19622s |
